# Effects of Unpredictable Perturbation Training on a Split-Belt Treadmill on Physical Performance in Older Adults: A Randomized Controlled Trial

**DOI:** 10.3390/geriatrics10010023

**Published:** 2025-02-07

**Authors:** Kap-Soo Han, Myoung-Hwan Ko

**Affiliations:** 1 Research Institute of Clinical Medicine of Jeonbuk National University-Biomedical Research Institute of Jeonbuk National University Hospital, Jeonju 54907, Republic of Korea; hanks@jbnu.ac.kr; 2 Department of Physical Medicine and Rehabilitation, Jeonbuk National University, Jeonju 54907, Republic of Korea

**Keywords:** rehabilitation, falls, unpredictable perturbation, balance, treadmill

## Abstract

**Background/Objectives**: This clinical trial aimed to determine whether perturbation-based gait training (PBGT) on a split-belt treadmill enhances balance and muscle strength in older adults, comparing its effectiveness with walking-only training on a treadmill. **Methods**: This single-center, prospective, single-blind (assessor), randomized controlled trial included 24 older adults from the Rehabilitation Center of Jeonbuk National University Hospital. Participants were equally divided into the PBGT and control groups. Both groups underwent 12 training sessions, three times a week for 4 weeks, for a total of 43 min per session. The outcomes, including the Five Times Sit-to-Stand Test (FTSST), Falls Efficacy Scale International, timed up-and-go (TUG) test, functional reach test, and lower-extremity manual muscle test scores, were measured at three time points: pre-training, post-training, and four weeks after training. **Results**: While there were no significant differences between the two groups, the PBGT group demonstrated significant improvements in its FTSST and TUG values. **Conclusions**: Unpredictable perturbation training on a split-belt treadmill can be safely performed by older adults and may serve as an alternative exercise method to enhance physical performance and balance ability for fall prevention.

## 1. Introduction

Falls are a major cause of death among older adults [1]. An increasing number of older adults have been reported to experience falls, which can be fatal because of severe injuries and complications [2]. Moreover, many older adults who have experienced falls tend to exhibit reduced spatial mobility in their daily lives due to fear of future falls [3]. The number of fall-related injuries is expected to increase alongside the continuous increase in the older adult population, leading to an increase in medical expenses, which poses a serious problem not only for the individuals concerned but also for society in general [4].

Regular exercise has been reported to prevent falls by increasing muscle strength and enhancing balance [5]. Enhancing balance and the performance of strengthening exercises have been shown to reduce fall rates in older adults by 21% [6,7]. To date, improving bodily function is one of the most effective methods found for preventing falls and reducing fall-related injuries. Repeated application of postural perturbations has been reported as a promising intervention for preventing falls by enhancing muscle performance and balance [8]. However, a validated fall prevention program or training system to implement this intervention is currently unavailable. Therefore, there is an urgent need to develop such programs and systems. Moreover, it is currently difficult for older adults to sustain a regular exercise regimen, and older adults who experience difficulties in planning these regimens may require expert assistance to access fall prevention programs.

Treadmill-based training has been used in studies involving various populations [9]. A study reported that postural perturbation training on a single-belt treadmill reduces the risk of falls [10]. Another study on postural perturbation training using a split-belt treadmill showed that the central nervous system adapts to postural perturbations by improving responsive stability through changes in compensatory gait length and trunk extension [11]. In addition, walking on a split-belt treadmill has been reported to reduce gait stability and symmetry [12] and considerably broaden the base of the gait compared to training on a single-belt treadmill [13]. A previous study on older adults with physical disabilities showed that perturbed walking exercise on a bilateral separate treadmill significantly improves balance and reaction time, as well as reduces the incidence of falls, compared to unperturbed walking exercise [14]. Moreover, significant improvements in static and dynamic balance were noted in a study using a wearable balance compensation system to improve the balance ability in patients with spinocerebellar ataxia [15]. However, studies specifically investigating postural perturbation training using a split-belt treadmill in healthy older adults are lacking. In addition, no studies have compared the effects of postural perturbation training between single-belt and split-belt treadmills. Treadmill gait training requires only a single running belt, and providing diversity in gait training using a single-belt treadmill is challenging. However, in this study, we used a developed fall prevention training method, wherein postural perturbations were induced by abruptly changing the walking speed of the left and right legs to enable the repetitive learning of postural perturbation conditions. They further developed an innovative fall-inducing training program using a split-belt treadmill. In a previous study that conducted perturbed gait exercises on disabled older adults using split belts, the treadmill exercise group experienced approximately 21% fewer falls than the control group, although without a significant difference [14]. To the best of our knowledge, this is the first study to perform fall prevention training methods using split-belt treadmills and compare control groups that performed daily life activities with perturbation training in healthy older adults. In this study, the perturbation stimulus was changed every 1–3 min using split belts to produce a more pronounced effect. This study aimed to compare the benefits of walking on a split-belt treadmill with unpredictable perturbations and on a treadmill with walking-only training for physical performance in older adults.

## 2. Materials and Methods

### 2.1. Study Design

This single-center, prospective, single-blind (assessor), randomized controlled trial was conducted at the Rehabilitation Center of Jeonbuk National University Hospital. This study was approved by the Institutional Review Board (IRB No.: 2019-03-053, Approval date: 6 September 2019). Written consent was obtained from each participant after the purpose of this study and the potential harms associated with participation were fully explained to them. This study is registered at the Clinical Research Information Service, under the direction of the Korea Centers for Disease Control and Prevention (Registration No.: KCT0004604).

### 2.2. Participants

This study was conducted with older adults (≥65 years of age) of both sexes. The inclusion criteria were the following: (i) ability to walk independently; (ii) ability to understand this study’s purpose and procedure; and (iii) provision of written informed consent to participate. The exclusion criteria were the following: (i) upper or lower-extremity disabilities due to paralysis or musculoskeletal disorders and (ii) recent surgery or fall event within the last year.

### 2.3. Randomization

Randomization was performed by using the randomization table utilization site, www.randomization.com, randomly assigning the control group and the experimental group in a 1:1 ratio, with a block size of 2. The table was prepared by one person not involved in this study, before the first subject’s screening, and it was made available to researchers when the subject was assigned a registration number. The assessors collecting study data were unaware of group assignments throughout the trial.

### 2.4. Sample Size

The sample size was set to 24 participants (12 per group), as calculated by Eggenberger et al. [16] Based on expected differences of 3.5 and 3.805 in the mean and standard deviation, respectively, and between pre-test and follow-up Falls Efficacy Scale International scores, the statistical significance was set to α = 0.05, power 1-β to 0.8, and drop-out rate to 20% of the treadmill walking with the simultaneous verbal memory training group (*n* = 17) and the only treadmill walking group (*n* = 15).

### 2.5. Intervention

**Figure 1:**
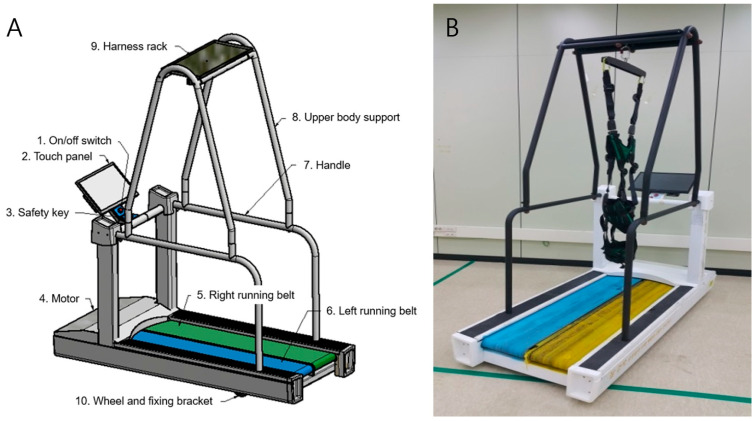
Split-belt treadmill (WALK WELL™) device. (**A**) Components of the split-belt treadmill. (**B**) This is a walk-training device used for rehabilitation exercises in which an appropriate velocity (0.3–3 km/h) can be set according to the conditions of individual patients. The device consists of two split belts that enable different velocity training simultaneously on each side.

All participants were randomly assigned to the control or perturbation-based gait training (PBGT) group. Both groups performed 12 sessions on a split-belt treadmill (WALK WELL™, IST Co., Ltd., Jeonju, Republic of Korea) (Figure 1). In the PBGT group, each belt was set to rotate at different velocities, whereas in the control group, each belt was set to rotate at the same velocity. This device is a walk-training device used for rehabilitation exercises, which can be set to an appropriate velocity (0.3−3 km/h), depending on the patient’s condition. Additionally, this device consisted of two split belts that allowed simultaneous training at different velocities on each side.

The PBGT group performed fall prevention gait training for 43 min (20 min walking + 3 min rest + 20 min walking) on a split-belt treadmill at varying speeds and time points on the left and right belts to induce postural perturbations. One session was divided into two phases. In the second phase, the velocity of both sides was reversed from that of the first stage. Participants walked at 2 km/h at the beginning of each phase. In the first and second weeks, the velocity changed every 2 min. We changed the velocity by 25–40% of the base value in the first week and 25–50% in the second week. In the third and fourth weeks, the velocity changed every 2, 1, and 3 min. We changed the velocity by 25–40% of the base value in the third week and 25–50% in the fourth week. The control group performed fall prevention gait training at a single velocity for 43 min (20 min of walking + 3 min of rest + 20 min of walking). Both groups trained three times a week (43 min per training session) for a total of 12 training sessions over a 4-week period. All measurements were conducted at three time points: evaluation 1 (E1, pre-training), evaluation 2 (E2, after training), and evaluation 3 (E3, 4 weeks after training).

### 2.6. Outcome Measurement

The outcome measures were the Five Times Sit-to-Stand Test (FTSST), Korean version of the Falls Efficacy Scale International (KFES-I), timed up-and-go (TUG) test, functional reach test (FRT), and lower-extremity manual muscle test (MMT).

The FTSST is commonly used to assess lower-extremity muscle strength and balance control. This test measures the time it takes to stand up from sitting with folded arms [17,18]. Meanwhile, the KFES-I is a 16-item self-report questionnaire used to assess the fear of falling experienced during various activities, ranging from routine to instrumental activities of daily living, including simple social activities. Participants indicate their perceived fear in four stages. Older adults who have experienced falls often exhibit an increased fear of falling, leading them to avoid routine and social activities, which further elevates the risk of falls. Consequently, assessing their fear of falling is crucial for effective fall prevention efforts [3].

The FRT assessed functional performance in terms of balance and flexibility in the frontal direction. Each participant was instructed to extend one arm forward horizontally, with their elbow straight and their shoulder flexed at a 90° angle while standing or sitting. The maximal distance from the third metacarpal bone was measured as the reach distance in centimeters. In a previous reliability study, involving 128 participants aged 20–80 years, the interrater reliability of the FRT was reported to be as high as 0.92 [19]. The TUG test is a functional mobility assessment tool with an interrater reliability of 0.94, which enables the quick assessment of a participant’s range of motion, mobility, and balance during the independent performance of ADLs [20]. The measurement consisted of standing up from a chair, walking for 3 m, turning around, and walking back to the chair. Walking and balance abilities were measured based on the time (seconds) required to complete the test. The TUG test is an effective screening tool for both functional mobility and the risk of falls in older adults [21]. An MMT device (Nicholas MMT gageTM) was used to measure the muscle strength required to move the hip (flexion, extension, abduction, adduction), knee (flexion, extension), and ankle (dorsiflexion, plantar flexion) joints.

### 2.7. Statistical Analysis

All statistical analyses were performed using SPSS version 23.0 for Windows (SPSS Inc., Chicago, IL, USA). Data are presented as the means (SD) for continuous variables and frequencies for categorical variables. For baseline differences (other than sex), the independent *t*-test was performed when the assumption of normality was satisfied; otherwise, the Mann–Whitney U-test was performed. Pearson’s chi-squared test was performed to compare the differences between groups for categorical variables according to demographics (sex). Outcome data were analyzed using repeated measures (RM) analysis of variance (ANOVA). The post hoc test was performed as planned with Bonferroni correction at a *p*-value of <0.05 for variables showing significant values. Statistical significance was set at a *p*-value of <0.05.

## 3. Results

### 3.1. Participants

**Figure 2:**
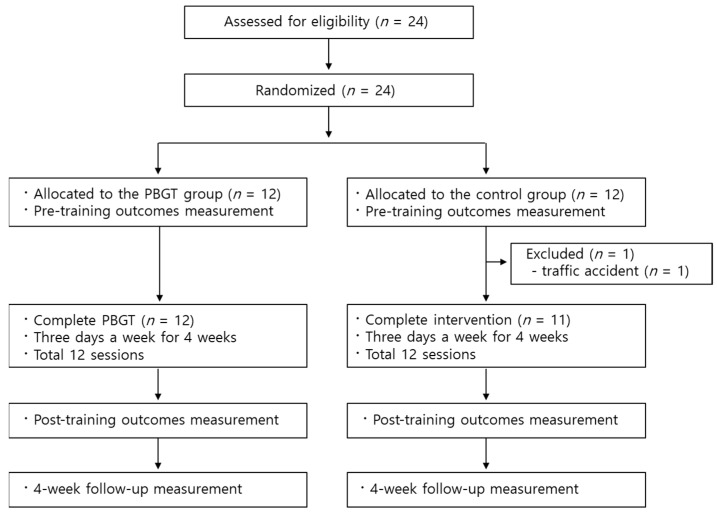
Participant flow diagram. Abbreviations: PBGT, perturbation-based gait training.

A total of 24 older adults (≥65 years) of both sexes were recruited between 7 August 2020 and 21 April 2021 and randomly assigned to two groups. One participant in the control group dropped out due to fractures resulting from a traffic accident, which made it impossible for them to continue participating in the clinical trial and training sessions. After excluding this participant, all others completed the treatment and evaluation (baseline, post-treatment, and 4-week follow-up evaluation) (Figure 2).

The demographic data for each group are shown in Table 1. No significant differences in mean age, sex ratio, or baseline outcomes were observed between the control and PBGT groups.

### 3.2. Outcomes

The FTSST and TUG exhibited significant variations in the time effect of RM-ANOVA (FTSST, *p* = 0.004; TUG, *p* = 0.063), but not in the group effect (FTSST, *p* = 0.515; TUG, *p* = 0.366) or the time × group effect (FTSST, *p* = 0.659; TUG, *p* = 0.593). For the FRT, there were no significant differences in the RM-ANOVA results. KFES-I was analyzed using the Friedman test (Table 2).

**Figure 3:**
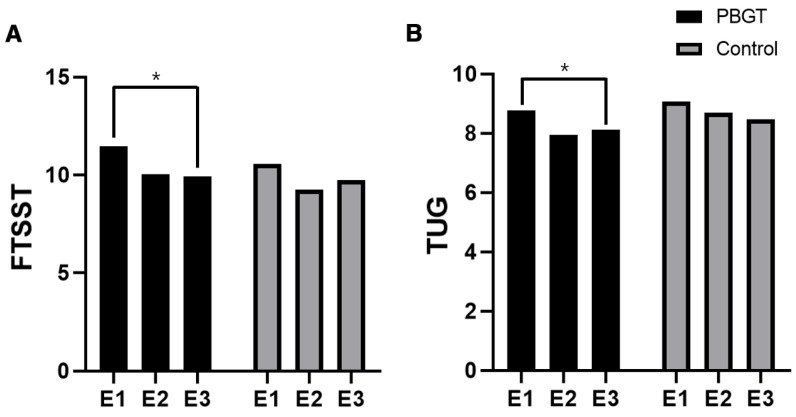
Results of outcome measures. * Within-group comparison *p* < 0.05. (**A**) The FTSST score, (**B**) TUG score.

In the intragroup comparison, the PBGT group showed a significant increase in its FTSST and TUG values (FTSST, *p* < 0.001; TUG, *p* = 0.011). The post hoc test of the PBGT group revealed significant differences between E1 and E2 (*p* = 0.013) and E1 and E3 (*p* = 0.010) in the FTSST. In the TUG, a post hoc test revealed significant differences between E1 and E2 of the PBGT group (*p* = 0.015) (Figure 3). There were no significant differences in the FTSST and TUG scores in the control group (Table 2).

Although the group effect did not reach statistical significance, there were notable improvements in the FTSST, KFES-I, TUG, and FRT values after the treatment (FTSST, ∆E2–E1_PBGT_: 1.40 ± 1.35 vs. ∆E2–E1_Control_: 1.36 ± 2.35; KFES-I, ∆E2–E1_PBGT_: 0.50 ± 1.00 vs. ∆E2–E1_Control_: 0.43 ± 1.04; TUG, ∆E2–E1_PBGT_: 0.84 ± 0.83 vs. ∆E2–E1_Control_: 0.61 ± 1.17; FRT, ∆E2–E1_PBGT_: −1.77 ± 2.41 vs. ∆E2–E1_Control_: −0.63 ± 3.19). No statistically significant improvements were observed in the lower-extremity MMT using the Nicholas MMT device.

E1, pre-training assessment; E2, assessment at the end of the training; E3, assessment 4 weeks after the end of the training; PBGT, perturbation-based gait training; KFES-I, Korean version of the Falls Efficacy Scale International; FTSST, Five Times Sit-to-Stand Test; FRT, functional reach test; TUG, timed up-and-go test.

A. The FTSST score changed for all participants. In the within-group comparison, a significant improvement was observed in the PBGT group but not in the control group (within-group comparison: *P*_PBGT_ < 0.001 vs. *P*_Control_ = 0.255). B. The TUG test score changed in all participants. In the within-group comparison, a significant improvement was observed in the PBGT group but not in the control group (within-group comparison: *P*_PBGT_ = 0.011 vs. *P*_Control_ = 0.679).

Abbreviations: PBGT, perturbation-based gait training; E1, pre-training assessment; E2, assessment at the end of training; E3, assessment 4 weeks after the end of training; FTSST, Five Times Sit-to-Stand Test; TUG, timed up-and-go test.

### 3.3. Safety Evaluation

We conducted safety evaluations to assess adverse events, including subjective and objective symptoms, as well as monitoring vital signs and collecting participant self-reports. Adverse events were defined as any new, undesirable medical findings that emerged during the study period. No adverse events related to the equipment or training sessions were reported by any participant throughout the study period.

## 4. Discussion

To the best of our knowledge, this is the first study to compare the effects of fall prevention training using a split-belt treadmill with perturbation to traditional walking using a single-belt treadmill in a group of healthy older adults. The objective of this study was to analyze the effects of fall prevention gait training using a split-belt treadmill (WALK WELL™) on enhancing lower-extremity muscle performance and balance in older adults. While no significant group differences were observed between groups, intragroup comparisons in the PBGT group revealed significant improvements in the FTSST and TUG tests. Furthermore, FTSST, KFES-I, TUG, and FRT measures improved when comparing pre- and post-treatment (E1-E2) results. These findings cautiously suggest that postural perturbation training on split-belt treadmills may enhance balance ability.

Falls are common among older adults because their bodies cannot appropriately adapt to unexpected situations. Training that involves unexpected perturbations to stimulate unconscious and reactive compensation stages is the most effective method of preventing falls [22]. Perturbation-based balance gait training improves stride and postural stability after post-postural perturbation through reactive step responses and rapid limb movement control for rebalancing in healthy older adults [23].

However, the mechanisms underlying the preventive effects of perturbations on falls are still not fully understood. Some studies have suggested that perturbation training works by inducing an immediate change in the cranial nervous system’s representation of the stable limit of the center of mass position, enabling a rapid response [24]. It has also been suggested that fear accelerates the development of adaptive synaptic pathways, thereby increasing the speed of learning through the fear induced by falls [25]. Studies have reported that perturbation using treadmills is the most effective perturbation-based balance training [26,27,28].

This study investigated the efficacy of postural perturbation training with a split-belt treadmill. The split-belt treadmill was developed to address the limitations of single-belt treadmills that do not consider differences in stride length between normal and affected sides when rehabilitating patients with unilateral gait impairments. Split-belt treadmills offer the advantage of enabling more targeted rehabilitation by adapting to differences in an individual’s right and left stride lengths, which may decrease the risk of falls. Furthermore, these treadmills are equipped with long bilateral handles along the running belt directions and an upper-body support system with a harness hanging from the overhead rack. These features help prevent secondary injuries which may occur during training on a split-belt treadmill. The velocity changed from section to section. After the speed changed, the participants slowed down as if they had tripped, but they managed to maintain their balance without anyone falling.

The FTSST is associated with lower-extremity muscle strength and is widely used to assess basic activities of mobility and independent walking based on movements necessary for various activities of daily living [18]. A previous study indicated that lower-extremity muscle strength enhances balance and thereby reduces the risk of falls. This finding supports the suitability of the proposed fall prevention gait training method used in our study [29]. Another study reported that the KFES-I score was significantly correlated with the modified Barthel index, an indicator of activities of daily living [30]. Therefore, the level of fear of falling might have been reduced in our study participants due to the postural perturbation training facilitated by speed control using a split-belt treadmill, which exerted a positive impact on their gait ability. The TUG test, a commonly employed measure of fall risk, was used to evaluate walking and balance abilities in our study [21]. The results indicate that continuous training is essential for enhancing walking and balance abilities. We observed that the PBGT group, which received training on a split-belt treadmill to induce postural perturbations, showed greater improvements in walking and balance abilities than the control group trained under single-belt conditions.

In this study, the FTSST and TUG test showed significant time effects in the PBGT group, probably due to improvements in balance ability and lower-extremity function through perturbation training. Since the FRT was relatively more related to flexibility, the effectiveness of treadmill training could be insignificant. From the KFES-I, perturbation training did not show a significant difference from classical training, but both exercises showed improved results, which were considered positive. While the results showed no significant differences between the groups, the use of a split-belt treadmill allowed for independent control of the left and right speeds and could be programmed according to individual user needs, demonstrating its potential for customized walking function in older adults. Furthermore, the significant changes observed in the PBGT group suggest that the inclusion of a complex pattern of varying walking speeds may have a positive impact on walking function in older adults.

This study has some limitations. This was a small-scale study conducted at a single center with a total of 23 participants (*n* = 23). However, our study addressed this limitation by employing the random assignment of participants to the PBGT and control groups and assessing a variety of physical functions during training on a split-belt treadmill. In addition, we did not perform imaging tests to evaluate the presence of brain lesions in the patients but instead checked for the presence of brain lesions according to the participants’ medical history. Further evaluations with larger sample sizes and participation from multiple centers are necessary to validate our findings. Nevertheless, the results of this study provide evidence supporting the effectiveness of postural perturbation training on a split-belt treadmill for enhancing balance ability and potentially preventing falls in older adults. We did not evaluate the cognitive function of participants, and worse executive functions have been reported to be related to falls in older adults [31]. Thus, future studies are warranted to conduct a cognitive test to understand the relationship with executive function. Furthermore, the split-belt treadmill used in this study has yet to be commercialized, and few hospitals or clinical centers can apply perturbation training due to the low availability of these devices.

## 5. Conclusions

The FTSST and TUG scores improved significantly in the PBGT group after training. Our study demonstrated that unpredictable perturbation training on a split-belt treadmill can be safely performed by older adults and may act as an alternative exercise method to enhance physical performance and balance for fall prevention in this population.

## Figures and Tables

**Table 1:** Demographic characteristics of the study participants.

	Control Group ^a^ (*n* = 11)	PBGT Group ^a^ (*n* = 12)	*p*-Value
Age	73.00 ± 3.29	74.08 ± 3.55	0.458 ^b^
Sex (male:female)	6:5	5:7	0.537 ^c^
FTSST	11.46 ± 2.90	10.55 ± 3.60	0.512 ^d^
KFES-I	18.17 ± 1.03	17.36 ± 0.67	0.064 ^b^
TUG	8.77 ± 1.61	9.06 ± 1.86	0.693 ^d^
FRT	17.56 ± 6.52	21.28 ± 5.92	0.168 ^b^

^a^ Data are shown as the mean ± standard deviation; ^b^ Analyzed by Mann–Whitney U-test; ^c^ Analyzed by Fisher’s exact test; ^d^ Analyzed by independent *t*-test; PBGT, perturbation-based gait training; KFES-I, Korean version of Falls Efficacy Scale International; FTSST, Five Times Sit-to-Stand Test; FRT, functional reach test; TUG, timed up-and-go test.

**Table 2:** Results of the outcome measures and comparisons between and within groups.

	PBGT ^a^				Control ^a^				PBGT vs. Control ^b^		
	E1	E2	E3	*p* ^c^ (*d*) ^d^	E1	E2	E3	*p* (*d*)	Time *p* (*d*)	Group *p* (*d*)	Time × Group *p* (*d*)
FTSST	11.46 ± 2.90	10.06 ± 2.07	9.94 ± 2.03	<0.001 (0.504)	10.55 ± 3.60	9.24 ± 1.72	9.73 ± 2.85	0.255 (0.128)	0.004 (0.230)	0.515 (0.020)	0.659 (0.020)
KFES-I	18.17 ± 1.03	17.67 ± 1.37	17.50 ± 1.09	0.061 ^e^ (0.255) ^f^	17.36 ± 0.67	17.00 ± 1.18	16.64 ± 1.03	0.087 ^e^ (0.204) ^f^	–	–	–
TUG	8.77 ± 1.61	7.94 ± 0.99	8.11 ± 1.08	0.011 (0.335)	9.06 ± 1.86	8.69 ± 1.17	8.47 ± 1.49	0.679 (0.092)	0.021 (0.169)	0.366 (0.039)	0.593 (0.025)
FRT	17.56 ± 6.52	19.33 ± 6.39	17.35 ± 5.95	0.242 (0.123)	21.28 ± 5.92	20.68 ± 5.62	20.40 ± 5.14	0.768 (0.026)	0.442 (0.038)	0.247 (0.063)	0.387 (0.044)

^a^ Data are shown as the mean ± standard deviation; ^b^ Analyzed by RM-ANOVA; ^c^ Post hoc: Bonferroni test (*p* < 0.05); ^d^ Cohen’s *d* for effect size (*d*); ^e^ Analyzed by the Friedman test; ^f^ Kendall’s W value for effect size (*d*).

## Data Availability

All data generated or analyzed during this study are included in this published article.
